# Effect of Multipath Laser Shock Processing on Microhardness, Surface Roughness, and Wear Resistance of 2024-T3 Al Alloy

**DOI:** 10.1155/2014/490951

**Published:** 2014-03-06

**Authors:** Abdulhadi Kadhim, Evan T. Salim, Saeed M. Fayadh, Ahmed A. Al-Amiery, Abdul Amir H. Kadhum, Abu Bakar Mohamad

**Affiliations:** ^1^Applied Science Department, University of Technology, Baghdad 10066, Iraq; ^2^Ministry of Education, Anbar, Iraq; ^3^Department of Chemical and Process Engineering, Faculty of Engineering and Built Environment, Universiti Kebangsaan Malaysia, Bangi, 43600 Selangor, Malaysia

## Abstract

Laser shock processing (LSP) is an innovative surface treatment technique with high peak power, short pulse, and cold hardening for strengthening metal materials. LSP is based on the application of a high intensity pulsed laser beam (*I* > 1 GW/cm^2^; *t* < 50 ns) at the interface between the metallic target and the surrounding medium (a transparent confining material, normally water) forcing a sudden vaporization of the metallic surface into a high temperature and density plasma that immediately develops inducing a shock wave propagating into the material. The shock wave induces plastic deformation and a residual stress distribution in the target material. In this paper we study the increase of microhardness and surface roughness with the increase of laser pulse energy in 2024-T3 Al alloy. The influence of the thickness of the confining layer (water) on microhardness and surface roughness is also studied. In addition, the effect of LSP treatment with best conditions on wear behaviors of the alloy was investigated.

## 1. Introduction

High-power lasers can be used to modify metallic surfaces. One of the examples of the surface modification techniques is the laser shock processing. Laser shock processing (LSP) is an innovative surface treatment, with which mostly a Q-switched Nd:YAG laser with short pulses of several nanoseconds and with a power density, in the pulse peak, of as much as several tens of GW/cm^2^ is used [1, 2]. The high magnitude stress wave generated by the interaction between the high-power, short pulsed lasers and the materials can produce plastic deformation. The plastic deformation caused by the shock wave produces compressive residual stresses at the sample surface. The increasing compressive residual stress value and depth would significantly improve the material's properties [3–5]. Laser shock processing can produce increases in metal surface hardness over the entire region of the laser irradiated area. The magnitude of surface hardening depends on the laser shock processing conditions, alloy type, and microstructure of the alloys. Microhardness increases with the LSP pulse energy. Hence, many of the proposed applications of LSP are aimed at improving the fatigue life, wear resistance, and corrosion resistance of the material through surface modification; surface roughness plays a highly important role on fatigue lives of alloys. The previous studies rarely involve the full investigation of surface topography about LSP treatment. Lu et al. investigated the surface qualities of LSP zones to the fatigue life of aluminum alloy [6]. They found that the fatigue life decreased with the increase of surface roughness. Aluminum alloy is widely used in aerospace and automotive industries due to its light weight and moderate strength. Considerable research has been carried out to examine the effects of LSP on the mechanical properties and fatigue lives of aluminum alloys. Most of the research showed that the mechanical properties and fatigue lives improved significantly for aluminum alloys due to the compressive residual stresses after LSP [7]. However, few studies have focused on the effects of LSP on the microhardness and surface roughness of aluminum alloy and its behavior with changing laser fluence and power density and other LSP parameters such as thickness of water layer above work piece. With the above background in mind, in this work we present a configuration and results in the LSP concept for metal surface treatments under water and irradiation 2024-T3 aluminum alloy spacemen's with laser wavelength 1064 nm. The objective of this work is to assess the feasibility of using laser beam to induce high compressive residual stress in 2024-T3 aluminum alloy specimens and the capability to penetrate deeper in specimens. Process parameters such as absorbent overlay and thickness of water layer varied as well.

## 2. Experimental Procedures

### 2.1. Experimental Set Up

**Figure 1:**
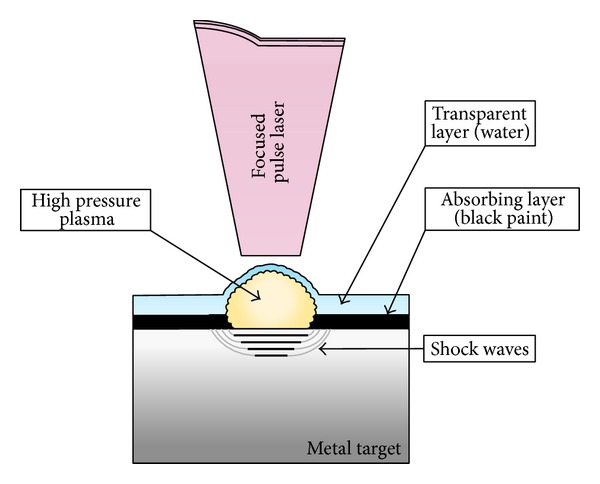
Schematic (LSP) metal target with absorbing, water layers and shock wave generation by laser beam.

The principle set up of laser shock processing (LSP) is shown in Figure 1. The metallic sample surface is to be treated firstly coated by an absorbing layer (black paint) and completely immersed in deionized water. The opaque overlay acts as a sacrificial material to avoid a thermal effect from the surface heating by laser beam and a thin layer of it vaporizes upon the absorption of laser energy [8, 9]. When the laser pulse with sufficient intensity (>GW cm^−2^) is directed onto the surface to be treated, it passes through the transparent overlay and strikes the sample. A short laser pulse is focused on the sample. Immediately the absorbing layer and a thin surface layer are vaporized and form plasma. The plasma continues to strongly absorb the laser energy until energy deposition. The rapidly expanding plasma is trapped between the surface of the sample and the transparent confining layer, creating high pressure (more than 1 Gpa) which propagates into the sample as shock waves exceed the Hogoniu Elastic Limit (HEL) of the metal material; plastic deformation then occurs in the surface layer [8, 10, 11]. The LSP process parameters that may be varied include the power density or fluence and height of transparent layer. In order to obtain the required pressure, a transparent overlay is used to confine the plasma expansion; in this work water is used. Water tends to confine the energy and increases the pulse pressure intensity against the base metal.

### 2.2. Samples Preparation

**Figure 2:**
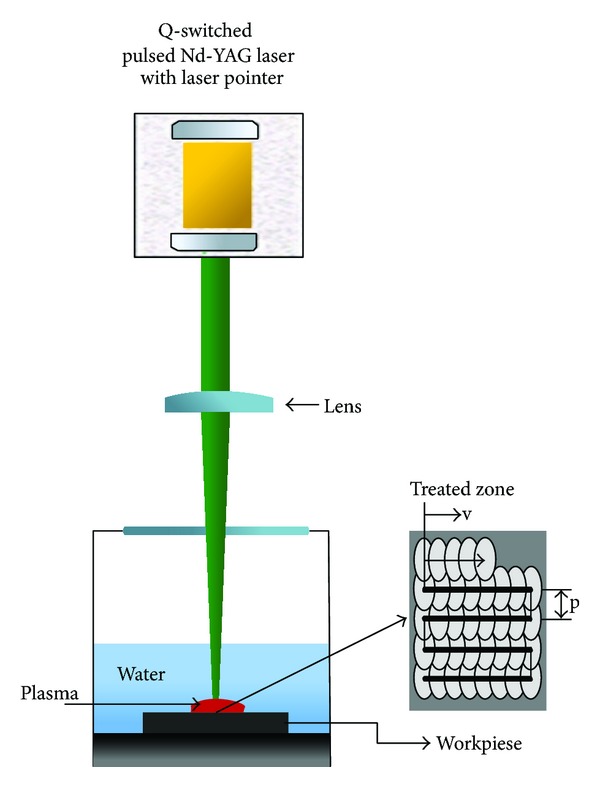
Schematic diagram of the experimental setup of (LSP) and the irradiation pattern on a sample.

The aluminum alloy 2024-T3 was machined as a rectangular shape with dimensions of 20 × 20 × 30 mm^3^ and the wear test samples into disk shape with a diameter of 2.4 mm and a thickness of 3 mm. The chemical composition of a 2024-T3 aluminum alloy sample was conducted by XRF as shown in Table 1. The samples were polished with metallographic (SiC) paper with different grades of roughness and polishing by diamond paste with lubricated liquid on cloth paper, followed by cleaning with distilled water and ethanol was used to degrease the sample surface and LSP experiments were conducted shortly after preparation. The experimental array is shown in Figure 2.

**Figure 3:**
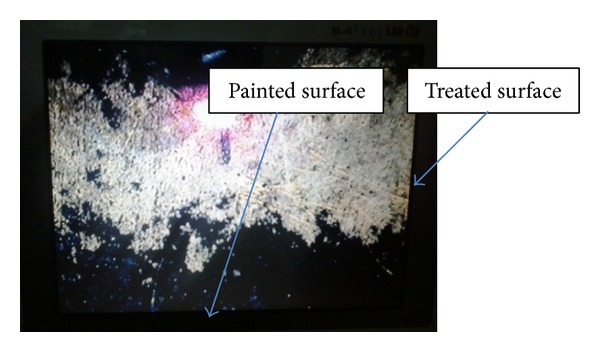
Optical image showing the treated sample.

During LSP, the shock waves were induced by a Q-switched Nd-YAG repetition-rate laser with a wavelength of 1064 nm, a pulse duration of 10 ns at FWHM, and a diameter of 1 mm, and the laser energy varied from 500 mJ to 1 J. A water curtain with different thicknesses was used as the transparent confining layer and the black paint with a thickness of 20 *μ*m was used as an absorbing layer to protect the sample surface from thermal effect as shown in Figure 3. During LSP impact, the laser beam was perpendicular to the sample surface during experiment, and the water layer was replaced after each line impact to keep the water purity and to avoid water bubble formation or the impurities coming from the material ablation due to laser treatment [11]. The overlapping rate was 50% between two adjacent spots in order to ensure no blind area at the LSP shocked region as shown in Figure 3 and the pulse density was 266 pulse/cm^2^. All processing parameters used in LSP were tabulated in Table 1.

### 2.3. Composition Analysis

All samples that have been used in this work were analyzed by using X-ray fluorescence (XRF) technique to identify the chemical composition.

### 2.4. Measurements of Microhardness

The microhardness of aluminum alloy 2024-T3 samples were measured by using the Vickers hardness method with “Digital Micro-Vickers Hardness Tester TH714.” The measurements were conducted with a load of 200 g. Three measurements were taken and then averaged.

### 2.5. Surface Roughness

Roughness measurement of all specimens was performed using “Digital Surface Roughness Tester TR-220.” The measurements of mean arithmetic roughness (*R*_*a*_) were conducted with different laser energy, different thickness water layer, and before and after LSP treatment. In a case of treated specimens, direction of roughness measurement was parallel to LSP swept direction to establish surface profiles in the longitudinal direction, by computing the average values of *R*_*a*_. The measuring specimen length was fixed at 10 mm for all specimen.

### 2.6. Wear Test

The wear rate has been calculated by the weighing method in which the wear testing system consisted of hard disc made from material type CK-45. Hardness = 54 ± 5 HRC, and the speed of motor was 900 rpm. The sample has been subjected to a direct attachment with the rotating disk for 10 min and under the normal load of 1 N. All samples were weighed before and after running by using a sensitive electronic balance of accuracy of 10^−4 ^g.

## 3. Results and Discussion

### 3.1. Composition Analysis

The chemical analysis of specimens was conducted by using XRF technique and tabulated as shown in Table 2.

### 3.2. Microhardness Results

**Figure 4:**
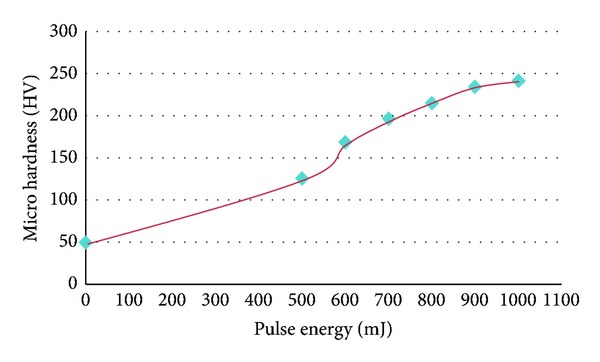
Microhardness [HV] of 2024-T3 aluminum after different LSP pulse energies.

The microhardness measurements of the specimens surface after laser treatment versus with LSP pulse energies are shown in Figure 4. The microhardness values increase with LSP pulse energy. At the laser pulse energy of 1 J, the microhardness increased from 49.7 HV before LSP to 241.6 HV after LSP at the impact center. This behavior is due to the stress induced by the shock wave. After LSP, severe plastic deformations of specimen microstructure occurred resulting in high density dislocations, leading to the formation of dislocation cells and pinning of dislocation. When LSP pulse energy increases, the grain is further refined Therefore, after LSP, the surface microhardness increases mainly due to dislocation strengthening and grain refinement; this behavior agrees with [12].

**Figure 5:**
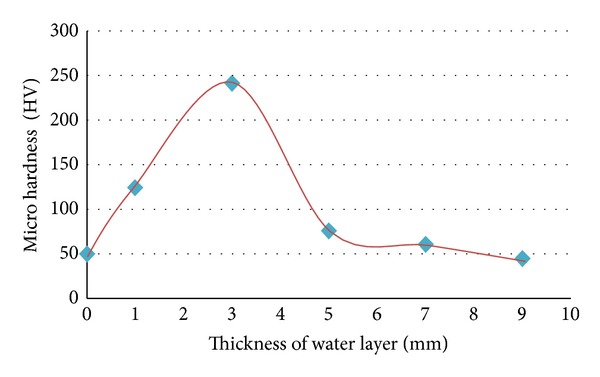
Microhardness [HV] of 2024-T3 aluminum.

Microhardness values of 2024-T3 Al vary with the thickness of water layer above the target at pulse energy of 1 J as shown in Figure 5 can note that the microhardness values increased from 49.7 HV to 241.6 HV at the water layer of 3 mm. When the thickness layer was less than 3 mm, the microhardness increased from 49.7 to 124.3 HV; this behavior is due to the fact that the pressure of the laser induced plasma at first was little and the effect of the shock wave up to water surface generated an acoustic wave before completing absorption of plasma laser energy.

More than 3 mm thickness, the microhardness has decreased because the water absorption reduces laser intensity and absorbs the energy of the laser pulse at the surface. The optimum thickness of water layer gives maximum micro hardness was 3 mm at pulse energy of 1 J, and wavelength of 1064 nm. 

### 3.3. Surface Roughness

**Figure 6:**
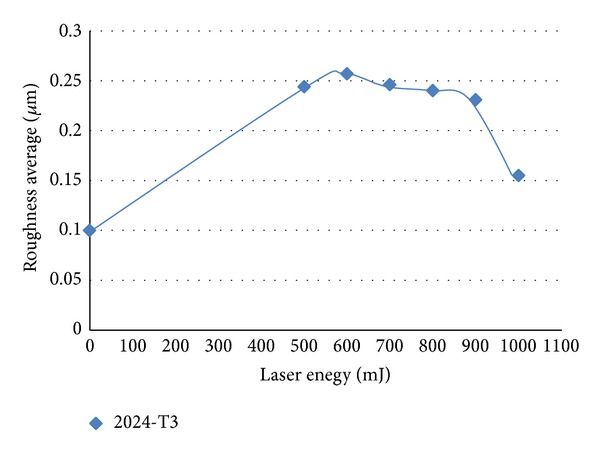
Surface roughness of treated region with different laser pulse energies.

**Figure 7:**
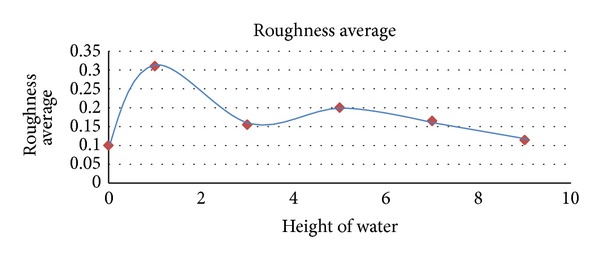
Surface roughness of treated region with different thicknesses of water layer.

The surface roughness of samples increased due to ablation and melting and it can be seen in Figure 6 that the roughness of the surface increased after LSP than before treatment. *R*_*a*_ (arithmetic average of the absolute values of all points of the profile) increased from 0.1 **μ**m before treatment to 0.155 **μ**m after laser irradiation with pulse energy of 1 J. The arising of the surface roughness of the specimen due to laser treatment indicates that the laser shot processing could be used after the elimination of cracks as a method of increasing the surface roughness area of an implant.  Figure 7 shows the higher surface roughness as a function of the confinement layer of 1 mm (water layer). The surface roughness decreases by increasing the thickness of the confinement layer; this behavior may be due to the fact that the roughness depends on the ablation rate which decreases by increasing the thickness of the confinement layer.

### 3.4. Wear Resistance

**Figure 8:**
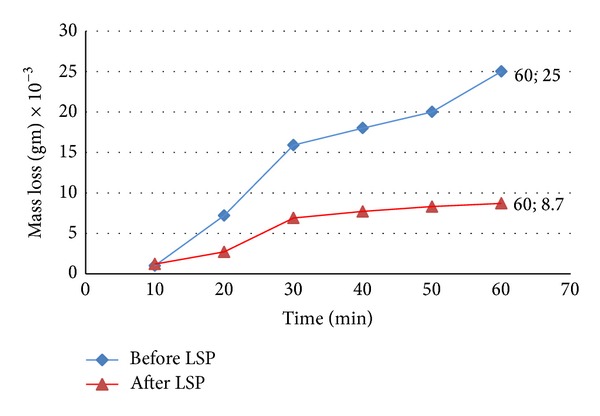
Variation of the mass loss on 2024-T3 specimens before and after LSP treatment with time.

**Figure 9:**
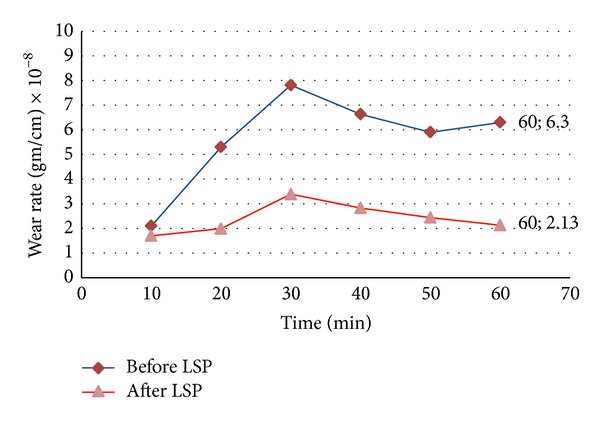
Variation of wear rate of 2024-T3 before and after LSP treatment with time.

Wear samples have been tested at the optimum condition: laser energy of 1 J and confinement layer thickness of 3 mm, which we have obtained from the study of surface roughness. The effect of surface roughness on wear resistance agrees with [13], where at high value of surface roughness the wear rate and weight loss increased.  Figure 8 shows the mass loss versus sliding time before and after LSP and it can be noticed the reduction of mass loss from 25 × 10^−3^ gm before LSP treatment to 8.7 × 10^−3^ gm after LSP at a sliding time of 60 min, in other words, the lack of the mass loss by 65% after LSP treatment. The variation of wear rate with sliding time can be shown in Figure 9. The results reveal that the smallest difference in wear rate between the treated and untreated samples occurs during the first 20 min interval. The LSP effects are observed clearly in the furthest material from the surface. The gradual decreases of wear-rate difference between the treated and untreated samples over the extended test duration may be related to wear regime transition from severe to mild. In other words the wear rate during the initial period remains at low value up to a later period. These results demonstrate that the severe wear rate during the early period of abrasion can be substantially reduced by properly applying LSP [13]. The grain size after LSP decreases in the micrometer regime near surface due to dislocation movement and then leads to a substantial hardening of materials and thus affect on the direct relation between grain size and wear resistance. As a result, the wear rate decreases, which means that the wear resistance will become increasingly denominated [14].

## 4. Conclusion

It has been demonstrated that LSP is an effective surface treatment technique to improve microhardness and wear resistance of 2024-T3 Al alloy. When pulse laser energy increased LSP treatment then the microhardness of the specimen increased too, and pulse laser energy (1 J) of LSP treatment the microhardness increased 4.8 times over the value before LSP treatment for 2024-T3 Al alloy. The surface roughness has increased from 0.1 *μ*m before LSP treatment to 1.55 *μ*m after treatment operation. The optimum confinement layer thickness (water layer) equals 3 mm for LSP treatment of 2024-t3 Al alloy at a wavelength of 1064 nm. Wear resistance increases for 2024-T3 Al alloy about 65% and wear rate decreased about 34% after LSP treatment at the optimum condition of pulse laser energy 1 J and thickness water layer 3 mm.

## Figures and Tables

**Table 1:** Laser shock processing parameters.

Laser energy (Joul)	0.5	0.6	0.7	0.8	0.9	1
Fluence (J/cm^2^)	**63.69**	**76.43**	**89.17**	**101.9**	**114.6**	**127.3**
Power density (GW/cm^2^)	**6.36**	**7.64**	**8.91**	**10.19**	**11.46**	**12.73**

**Table 2:** Composition of aluminum alloy specimens.

Composition	Si	Fe	Cu	Mn	Mg	Cr	Ni	Zn	Pb	Sn	V	Al
Percent (wt.%)	0.12	0.28	3.59	0.61	1.44	0.02	0.01	0.17	0.024	0.019	0.01	Bal.
